# Radiomics for Parkinson's disease classification using advanced texture-based biomarkers

**DOI:** 10.1016/j.mex.2023.102359

**Published:** 2023-09-05

**Authors:** Sonal Gore, Aniket Dhole, Shrishail Kumbhar, Jayant Jagtap

**Affiliations:** a Pimpri Chinchwad College of Engineering, Nigdi, Pune, Maharashtra, India; b Symbiosis Institute of Technology (SIT), Symbiosis International (Deemed University), (SIU), Lavale, Pune, Maharashtra, India

**Keywords:** Custom LBP method to extract advanced biomedical texture descriptors, Parkinson's disease, Radiomics, Local binary pattern, Support vector machine, Recursive feature elimination

## Abstract

Parkinson's disease (PD) is one of the neurodegenerative diseases and its manual diagnosis leads to time-consuming process. MRI-based computer-aided diagnosis helps medical experts to diagnose PD more precisely and fast. Texture-based radiomic analysis is carried out on 3D MRI scans of T1 weighted and resting-state modalities. 43 subjects from Neurocon and 40 subjects from Tao-Wu dataset were examined, which consisted of 36 scans of healthy controls and 47 scans of Parkinson's patients. Total 360 2D MRI images are selected among around 17000 slices of T1-weighted and resting scans of selected 72 subjects. Local binary pattern (LBP) method was applied with custom variants to acquire advanced textural biomarkers from MRI images. LBP histogram helped to learn discriminative local patterns to detect and classify Parkinson's disease. Using recursive feature elimination, data dimensions of around 150-300 LBP histogram features were reduced to 13-21 most significant features based on score, and important features were analysed using SVM and random forest algorithms. Variant-I of LBP has performed well with highest test accuracy of 83.33%, precision of 84.62%, recall of 91.67%, and f1-score of 88%. Classification accuracies were obtained from 61.11% to 83.33% and AUC-ROC values range from 0.43 to 0.86 using four variants of LBP.

• Parkinson's classification is carried out using an advanced biomedical texture feature. Texture extraction using four variants of uniform, rotation invariant LBP method is performed for radiomic analysis of Parkinson's disorder.
• Proposed method with support vector machine classifier is experimented and an accuracy of 83.33% is achieved with 10-fold cross validation for detection of Parkinson's patients from MRI-based radiomic analysis.
• The proposed predictive model has proved the potential of textures of extended version of LBP, which have demonstrated subtle variations in local appearance for Parkinson's detection.

## Graphical abstract

**Figure:**
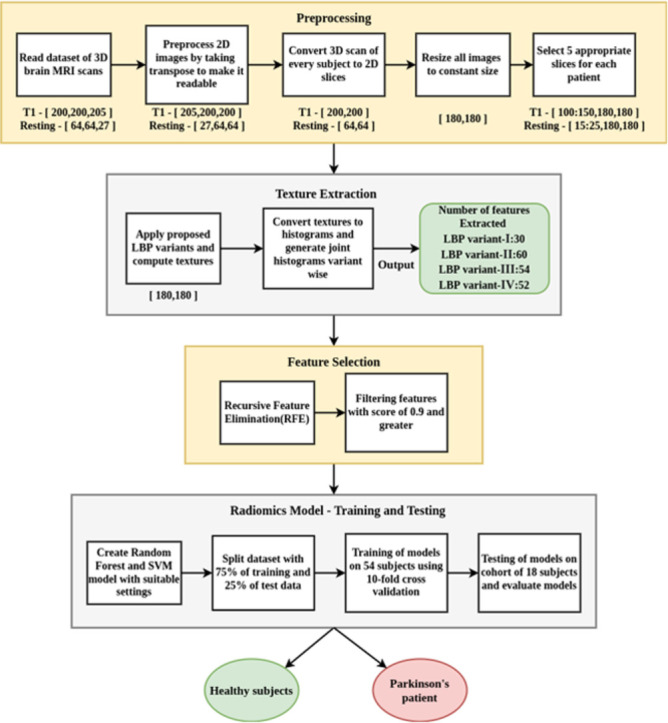

Specifications Table

**Table:** 

Subject area:	Computer Science
More specific subject area:	Medical Image Analysis using Machine Learning
Name of your method:	Custom LBP method to extract advanced biomedical texture descriptors
Name and reference of original method:	Method name: LBP variants: Variant-I, Variant-II, Variant-III, Variant-IV Reference of original method: Gore S, Jagtap J, Radiogenomic analysis: 1p/19q codeletion based subtyping of low-grade glioma by analysing advanced biomedical texture descriptors, Journal of King Saud University - Computer and Information Sciences. 34(10), 2022, pages 8449-8458. https://doi.org/10.1016/j.jksuci.2021.08.024
Resource availability:	Dataset link: http://fcon_1000.projects.nitrc.org/indi/retro/parkinsons.html

## Introduction

Parkinson's disease, referred to as PD is the second most common disease among old people. PD is found in among 1% of the world's population [1,2]. PD is a neurodegenerative disease with neurological symptoms like trembling of hands, slowness in movement, hard to speak, unable to swing hands. The main reason to cause disease are dead cells that trigger to form dopamine which is a chemical used for transmitting signals among neural cells [3,4]. Lack of dopamine may lead to break this communication due to which transfer of signals is not possible. The dopamine is present in brain areas such as ventral tegmental area, substantia nigra, and hypothalamus [5]. Some studies concluded that men have more risk with around 2% [1] whereas women have about 1.3% of risk. Early diagnosis of PD can help for immediate recommendation of medications, suitable treatment therapies in order to help to reduce the symptoms of disease and to improve quality of patients' life.

Multiple research works has explored different stages of PD which makes to understand disease more deeply [6]. In the initial stage, the person experiences just minor symptoms that do not interfere with regular activities. Only one side of the body has tremor and problems in other movement like change in posture, movement, and facial expressions. As stage advances, the symptoms start to worsen till 5^th^ stage of disease [6] like the effect of tremor on both sides of the body, high stiffness, motor skills related severe abnormalities, that makes routine lifestyle more difficult, time-consuming and more dependable on other persons [7].

The motor symptoms are one of the diagnostic markers for PD at very initial stage. Current diagnosis of PD depends on clinical evaluation of patients by medical experts that includes evaluation of patients' past reports, examination of their physical moments [8]. Neuroimaging techniques like functional magnetic resonance imaging (MRI), computer tomography (CT) scans are used to detail the diagnosis of PD [9]. Manual diagnosis of PD is susceptible to prolonged diagnosis and prognosis, inter-rater or intra-rater variability, etc. Hence, computer aided diagnosis (CAD) is needed with intelligent, advanced technological and computing solutions to auto-classify PD.

MRI provides comprehensive images of tissues using radio waves. Therefore, MRI-based radiomics is a useful technique to diagnose PD by understanding various imaging-derived biomarkers associated with PD. Since gadolinium-containing compounds act like an agent for paramagnetic contrasts, gadolinium enhanced T1-weighted MR scans provide best contrasts for those brain regions where the presence of PD is active [10]. Resting state functional MRI (rs-fMRI) scans are used to observe the networks of functional regions of the brain that tend to co-activate and these scans are generally carried out when a patient is not executing any specified task [11].

Artificial intelligence (AI) is a developing field nowadays that provides computational solutions in various fields of research with the help of deep learning and machine learning techniques. An ability to learn complicated functions by mapping the input to the output directly from data and by automatically learning features at several levels of abstraction allows an AI-based system to learn complex functions without relying entirely on human-crafted features. And health care systems are getting benefited with such algorithms to detect or analyse complex diseases which usually need manual intervention of medical experts in diagnosis. Therefore, it is more advantageous to diagnose PD with AI-driven solutions, that performs an analysis of radiomic features extracted from MRI phenotypes.

The proposed work specifically focuses on analysis of textures of MR brain images of healthy and PD controls, classifying those to normal and PD patient class. The existing techniques in the literature have reported PD classification techniques using MR image analysis, voice signals, statistical methods, that are applied on different clinical as well as research datasets [12], [13], [14], [15]. It was rarely found about study on the local-level texture descriptors, that helps in finding areas of the brain helpful to detect PD [16]. The current work has researched the significance of variants of local binary pattern (LBP) method [17] to derive local-level textures of glioma cancer patients for tumor subtyping at genomic level. Public dataset was collected from National Institute for Research and Development in Informatics, Romania. Neurocon datasets includes 43 subjects containing MRI scans of 27 PD patients and 16 control subjects. Tao Wu datasets includes 40 subjects containing 20 control subjects and 20 PD patients. Supervised machine learning classifiers such as SVM and random forest are barely implemented to analyse LBP-derived textural patterns, that are extracted using LBP variant-I, variant-II, variant-III and variant-IV.

## Literature review

Parkinson's is a second prevalent neuro-degenerative disorder, especially among old people [18]. The risk of disease is more in men (around 2%) as compared to women (about 1.3%) [19]. It is very crucial to detect the disease in early stage for immediate recovery, otherwise there is a major threat to suffer for a lifelong with progressive and more complex symptoms and impact. It is primarily characterized by presence of motor features in combination like tremor, rigidity in movements, etc. and non-motor symptoms are generally observed in later stage of disease. Currently, reliable test is not available with existing pathology for PD diagnosis in its early stage.

In last few years, AI-based solutions are being gradually accepted in automated diagnosis of PD where the researchers have presented various methods using artificial neural networks (ANN), deep learning, supervised or unsupervised machine learning, etc [20,21]. for detection purposes. The inclusion of AI-based auto-classifiers in PD diagnosis seems to be an ever-increasing need. Many studies have used machine learning approaches to detect PD due to their precise results. *K*-nearest neighbors (*k*-NN), SVM, logistic regression models, etc. were used for classification work. Precision, recall, sensitivity, specificity, accuracy, f1 score, AUC-ROC, etc. were majorly computed to evaluate the performance of models [22].

Even, different kinds of data were used for PD analysis, that includes speech data, voice data, conventional and functional MR imaging scans, etc. With great advancements in feature detectors, voice-based signals have been used for diagnosis and prediction of Parkinson's using static and dynamic speech features [23]. The amount of articulation transitions and the trend of the fundamental frequency curve are considerably different between PD patients and normal subject due to strange, uncontrolled, unvoiced sounds in PD patients. Bidirectional long-short term memory (LSTM) model is used to auto-capture time-series dynamic aspects of a speech signal for the purpose of identifying PD and it has achieved the maximum classification accuracy of 73.46%. Moreover, the voice signals were analysed using 195 recordings extracted from each among 31 patients, that are taken from University of California Irvine (UCI) repository [1]. Multilayer perceptron (MLP) and SVM methods were used for the principal diagnosis of PD, that has yielded performance accuracy of 92.3% and 93.3% respectively. Features such as baseline features, time frequency features, vocal fold features were used for analysis of acoustic signals to classify PD patients and healthy control subjects [24]. The set of 752 features of acoustic signals extracted from 252 subjects from UCI database were analysed with naive bayes classifier, that has achieved performance accuracy of 78.97%. In 2019, the work by Shamrat et al. has obtained a highest accuracy of 100% using SVM, second highest performance with an accuracy of 97% using logistic regression, whereas 69% score was achieved using k-NN for dissecting Parkinson's disease datasets of audio recordings [25].

The research work by Lawton et al. has validated subtypes of PD with two large independent cohorts of individuals newly diagnosed with Parkinson's disease by performing *k*-means based cluster analysis [26]. The researchers identified four clusters, first, an asymmetrical motor disease with poor olfaction, cognition, and postural hypotension; second, mild motor and non-motor disease with intermediate motor progression; third, severe motor disease with an intermediate motor progression, poor psychological well-being, and poor sleep; and last, slow motor progression with tremor-dominant, unilateral disease. The study by Vatsaraj et al. has used the handwritten images or drawings in diagnosis of PD by detecting the motor symptom of trembling of the hands, that generally exists in PD patients [27]. Accuracies of 73.05% and 73.33% were acquired by SVM and *k*-NN. Even though, subject experts were able to detect PD either from symptoms, or from audio/videos signals or from drawings images, it was recently observed that MRI scan images also play important roles to understand disease in less time and more effectively. Hence, many recent studies have worked to enhance the performance score for PD diagnosis using various imaging scans.

The method by Lavalle et al. relied on the use of voxel-based morphometry (VBM) on magnetic resonance images to identify the regions of interest within grey matter where dopamine-producing nerve cells have died. T1-weighted MR images from PPMI were chosen for this study that contain 226 MRI scans of PD men, 86 images of male controls, 104 images of female patients, and 64 images of female patients without PD. The study concluded that men have the best detection performance with 99.01% accuracy as compared to women [28]. Few research works have been conducted on texture, statistical or morphological feature analysis of volume of interest (VOI) or region of interest (ROI) drawn from subcortical regions of brain [29], [30], [31], [32], [33]. The study was carried out by segmenting eight subcortical structures using atlas-based segmentation. Top 20 features were selected and further analysed using ANN, XGBoost, SVM, and random forest classifiers. ANN has acquired best score with 95.3% accuracy [34]. The 3D texture analysis of substantia nigra has accurately differentiated PD subjects from healthy subjects [35]. The structural changes have been determined by analysing the gray level dependency for PD discrimination as reported by Sikio et al. [36]. Low frequency fluctuation based radiomic models were constructed using SVM classifier, that extracted first and high order radiomics features to discriminate the Parkinson's patients [37,38]

As development happened in medical fields, recent works have used deep learning methods for detecting Parkinson's using imaging scans. Convolutional neural network (CNN) architectures extract meaningful patterns from MRI images and are able to detect hidden features which were not possible by traditional machine learning methods. CNN architecture was presented to analyse T2-weighted MRI images extracted with mid-brain slices of 500 patients, that has obtained better accuracy (of 96%) as compared to machine learning classifiers [39]. A 3D CNN model was developed with 12 convolutional layers to analyse T1-weighted MRI scans of total 406 subjects (203 with PD and 203 healthy subjects) available with Parkinson's Progression Markers Initiative (PPMI) database. It has yielded an accuracy of 95.29%, f1-score of 0.93, recall of 0.94, precision of 0.92, and ROC-AUC score of 0.98 [35]. In other study, an accuracy of 88.9% was obtained by employing CNN model, which has improved the performance over popular AlexNet architecture [40].

It was found that multiple works have utilised voice signals, imaging scans, handwritten drawings, acoustic data in order to analyse and diagnose PD. But very few works are proposed on acquiring exact textural patterns from the images. The prediction accuracy can be improved by using pattern techniques with local-level texture extraction methods, that helps to understand the subtle differences in PD discrimination from healthy controls.

## Materials and methods

### Patient characteristics and data preparation

The data is publicly accessible and it is retrieved from National Institute for Research and Development in Informatics (NIRDI), Romania [41]. It contains T1-weighted and resting state MR scans of subjects with Parkinson's disease and without Parkinson's (i.e., healthy subjects). The dataset is divided into two parts on the basis of different acquisition parameters of MRI scanner i.e., Neurocon and Tao Wu. The Neurocon dataset consists of MRI scans of 27 Parkinson's subjects and 16 normal subjects whereas Tao Wu consists of MRI scans of 20 patients with Parkinson's and 20 healthy subjects. T1-weighted scanning was done using the magnetization prepared - rapid gradient echo (MPRAGE) sequence. 1.5 Tesla Siemens Avanto MRI scanner was used to capture scans for Neurocon dataset. And 3 Tesla scanners of Siemens Magnetom Trio were used for the Tao Wu MRI dataset. The resting state scan consists of 27 slices per brain. And the T1-weighted scan consists of 205 slices of brain. Among 83 subjects, 11 subjects were excluded due to incomplete slices and small size of the MRI volume. The slice images whose size was less than 180 × 180 pixels were excepted. Finally, 72 subjects (39 PD patients and 33 healthy subjects) were included in experimentation for PD detection.

**Fig. 1:**
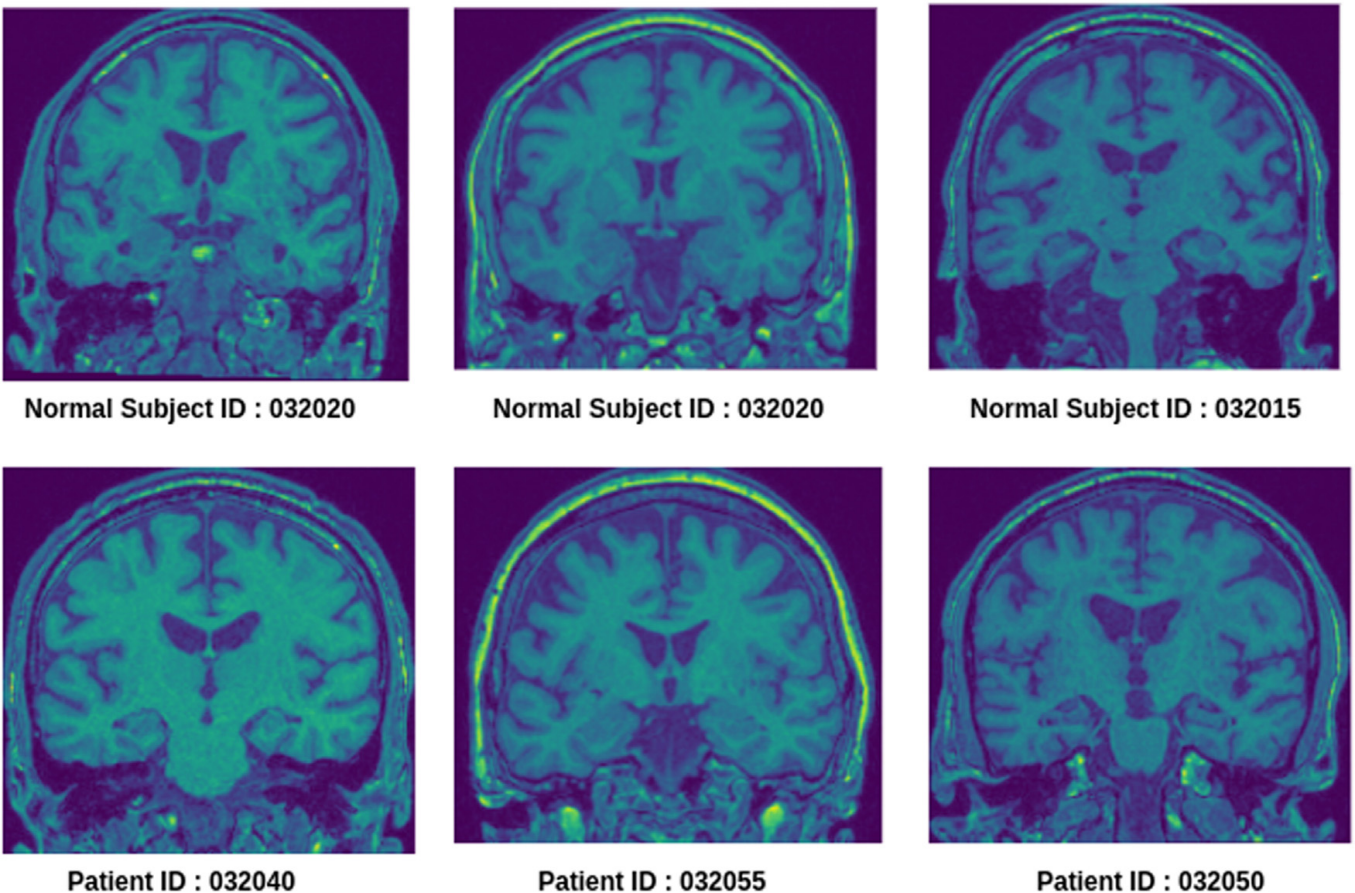
Sample images of 130^th^ slice of T1-weighted MRI (coronal plane) of healthy subjects and Parkinson's patients.

Sample MRI images of a healthy control and Parkinson's subjects are shown in Fig. 1. It signifies a minute visual difference in locations of dopamine shown as yellow spots in certain regions of the brain such as substantia nigra.

**Fig. 2:**
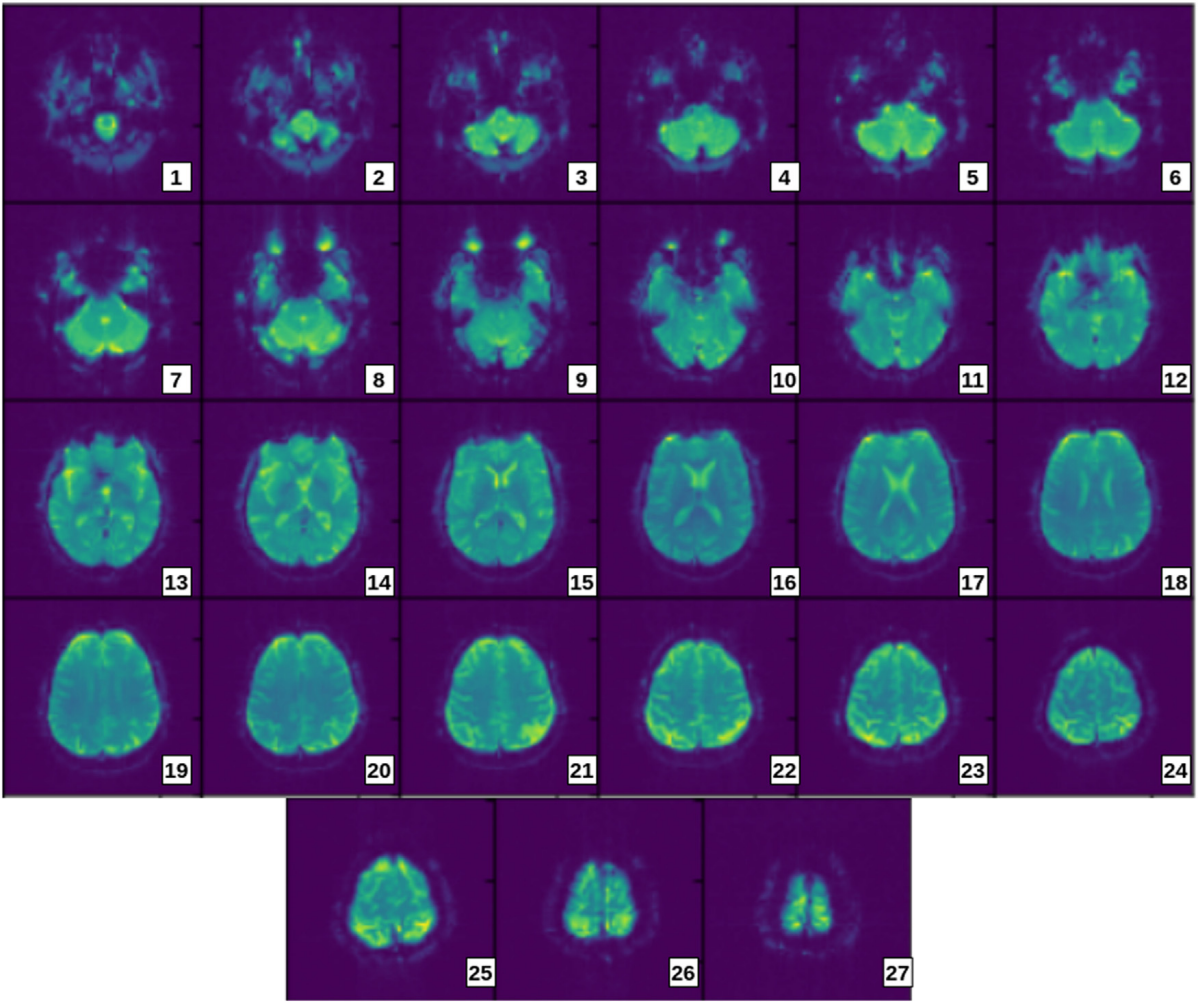
Axial 2D slices extracted from rs-fMRI volume of sample Parkinson's patient.

The set of 2D images shown in Fig. 2 are extracted from a 3D rs-fMRI scan of sample Parkinson's patient (patient ID-032033). The figure helps to visualise the flow and pattern of 2D slices of a Parkinson's subject. It also gives an idea for manual selection of useful slices based on size and difference in colours.

### Methodology

**Fig. 3:**
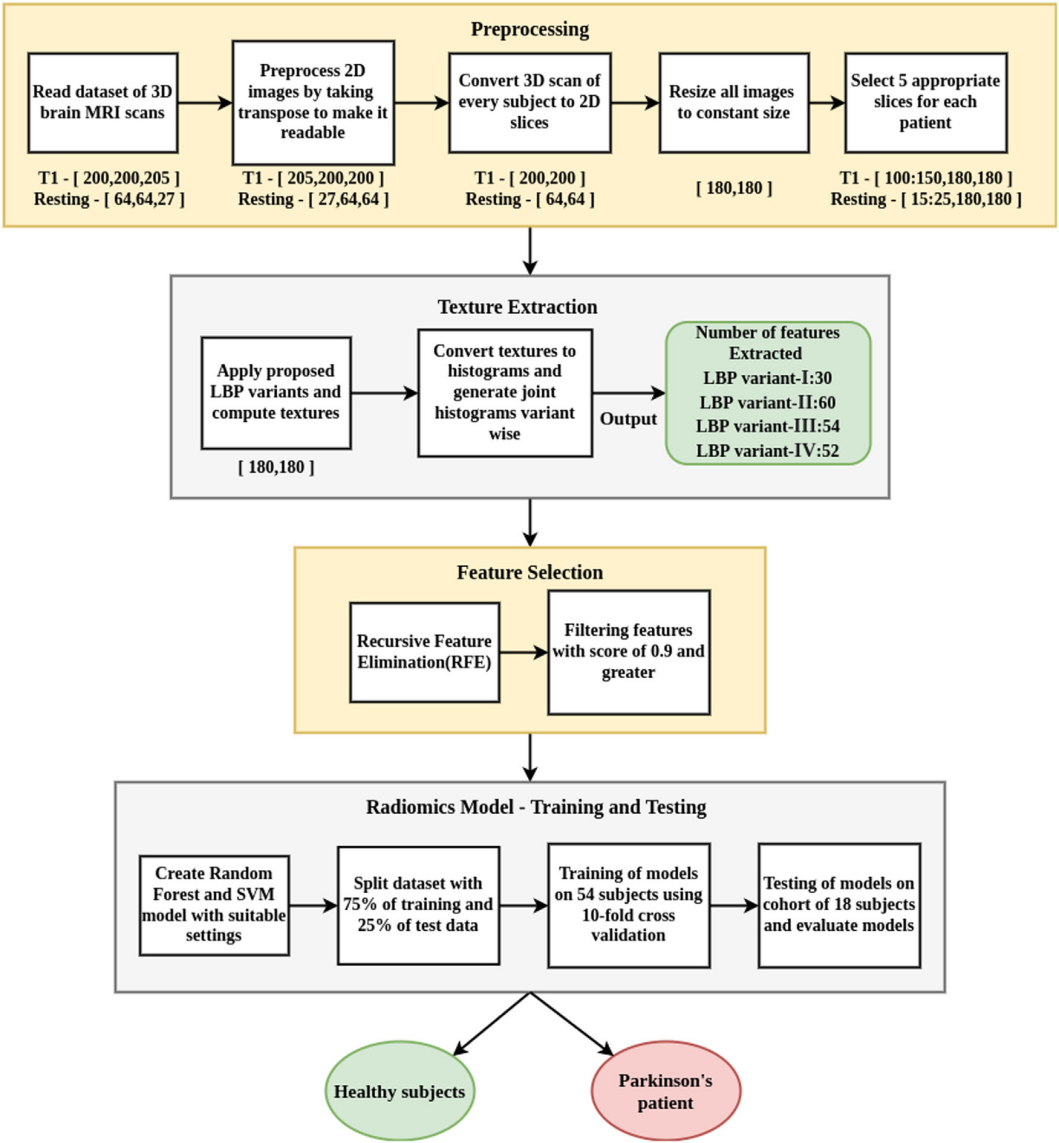
Flow diagram of radiomics model.

A flow diagram of proposed radiomics methods using LBP-derived textures and using machine learning algorithms is shown in Fig. 3. It contains four major steps, that includes 1) pre-processing of data, 2) extraction of local textural patterns using advanced LBP variants, 3) feature selection using recursive feature elimination, 4) 10-fold cross validation of model for training and testing of model on independent cohort using random forest and SVM methods.

1. Pre-processing: In order to prepare the data for further processing, minimal pre-processing steps are carried out as follows. 1) Each 3D MRI scan is sliced into 2D images using the NiBabel tool. 2) Further, those images are resized to 180 × 180. 3) The slices containing maximum extent of brain region (especially substantia nigra) are manually selected per subject by analysing the size of the centre region of the brain. Accordingly, five appropriate slices are chosen for each subject. Due to the huge size of data, only certain five slices of the brain are selected in the range of slice number 100 to slice number 150 with a step of 10 i.e., 100^th^, 110^th^, 120^th^, etc. images were selected as these were having maximum potential of accuracy. In total, dataset of 72 subjects resulted in to 360 images.
2. Texture Extraction: Local level textural patterns are extracted using four LBP variants proposed by Gore et al. [17]. Those variants contain variant-I using 8-bit LBP method applied on 5 × 5 neighborhood, variant-II using 8-bit LBP applied on 7 × 7 neighborhood, variant-III using 16-bit LBP applied on 7 × 7 neighborhood, variant-IV using 24-bit LBP applied on 7 × 7 neighborhood. Textures are generated using rotation-invariant uniform LBP method and then appropriately converted to LBP histograms. These histograms are combined into single feature vector variant wise and fed further for model training.
3. Feature Selection: Feature reduction and selection is done using the RFE algorithm, that eliminates features based on elimination rank and importance score of features.
4. Model Training and Testing: LBP variant-based radiomic model is cross-validated using random forest and support vector machine methods to classify whether a subject has Parkinson's or not. The model was trained on 75% of the whole dataset using 10-fold cross validation and the remaining 25% subjects were used for testing with evaluation parameters like accuracy, f1-score, AUC-ROC, precision and recall.

### Extraction of advanced textures using LBP variants

We have applied four custom variants [17] of LBP which were applied on multiple scales with neighborhood size of 5 × 5 and 7 × 7.

1. **Variant-I using 8-bit LBP applied on 5 × 5 neighborhood**

**Fig. 4:**
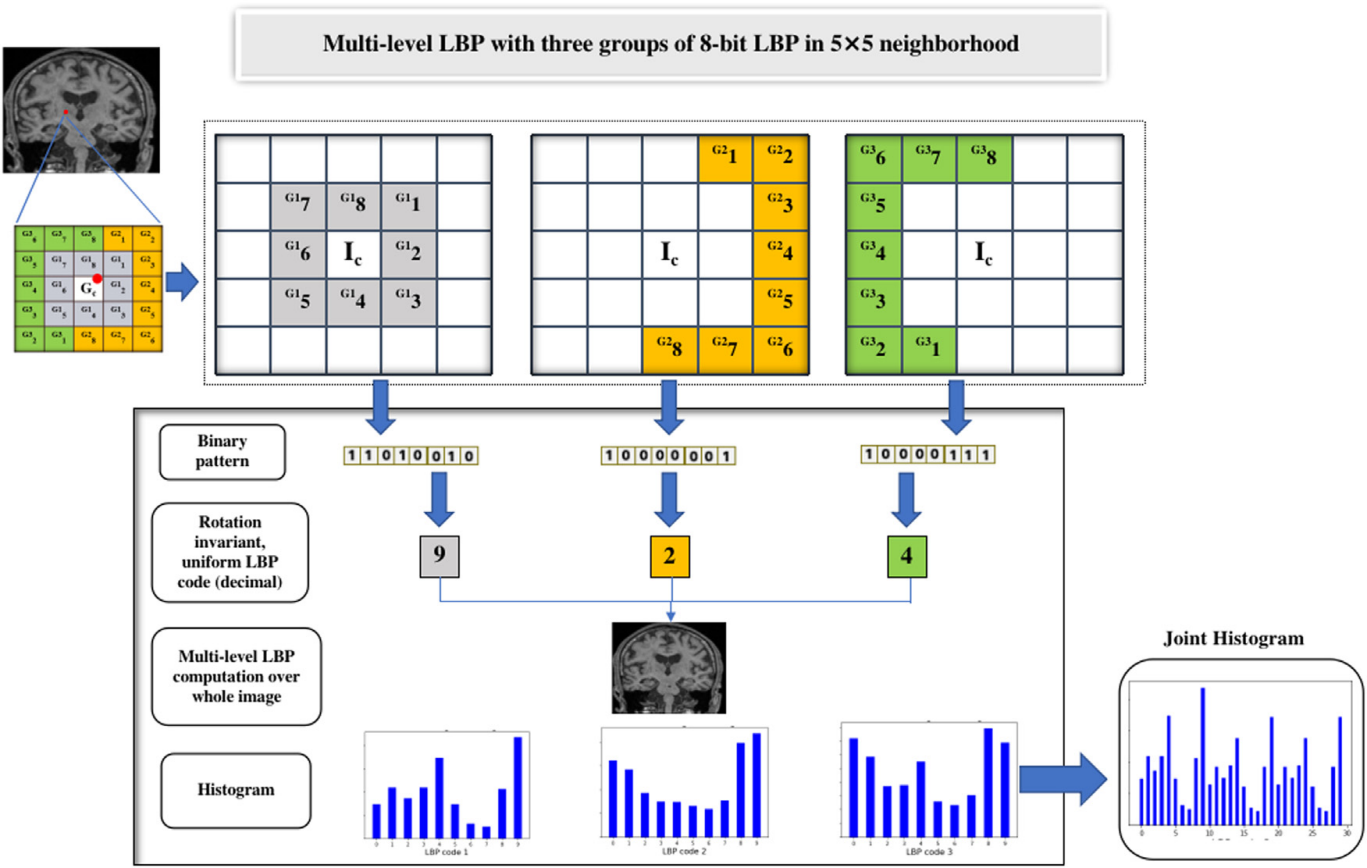
Computation of 8-bit LBP codes using LBP variant-I.

This variant is applied on whole image pixel wise with 5 × 5 neighborhood, that consists of 25 pixels in neighborhood matrix; out of which 24 neighboring pixels of each center pixel are grouped into 3 sets of 8 pixels each i.e., G1, G2, G3, which is also equivalently termed as regions (i.e., region-1, region-2, region-3) [17]. Each of these groups is then further computed into LBP histogram and further to a joint histogram after combining resultant 3 LBP histograms. The histograms are computed by converting each LBP image into bins of size 10, which are numbered based on LBP codes ranging from 0 to 9. This has resulted into feature vector of size 30 per slice and eventually resulted into feature set of size 150 for five selected slices of every subject. The working of LBP variant-I, that computes three 8-bit LBP codes, 3 LBP histograms, and finally a joint histogram, is shown in Fig. 4.

2. **Variant-II using 8-bit LBP applied on 7 × 7 neighborhood**

This variant is applied on whole image pixel wise with 7 × 7 neighborhood, that consists of 49 pixels in neighborhood matrix; out of which 48 neighboring pixels of each center pixel are grouped into 6 sets of 8 pixels each i.e., G1, G2, G3, G4, G5, G6, which is also equivalently termed as regions (i.e., region-1, region-2, region-3, region-4, region-5, region-6) [17]. Each of these groups is then further computed into LBP histogram and further to a joint histogram after combining the resultant 6 LBP histograms. The histograms are computed by converting each LBP image into bins of size 10, which are numbered based on LBP codes ranging from 0 to 9. This has resulted in a feature vector of size 60 per slice and finally resulted in a feature set of size 300 for five selected slices of every subject.

3. **Variant-III using 16-bit LBP applied on 7 × 7 neighborhood**

This variant [17] is applied on whole image pixel wise with 7 × 7 neighborhood, that consists of 49 pixels in neighborhood matrix; out of which 48 neighboring pixels of each center pixel are grouped into 3 sets of 16 pixels each i.e., G1, G2, G3, which is also equivalently termed as regions (i.e., region-1, region-2, region-3) [17]. Each of these groups is then further computed into LBP histogram and further to a joint histogram after combining resultant 3 LBP histograms. The histograms are computed by converting each LBP image into bins of size 18, which are numbered based on LBP codes ranging from 0 to 17. This has finally resulted into feature vector of size 54 per slice and eventually resulted into feature set of size 270 for five selected slices of every subject.

4. **Variant-IV using 24-bit LBP applied on 7 × 7 neighbourhood**

This variant is applied on whole image pixel wise with a 7 × 7 neighborhood that consists of 49 pixels in neighborhood matrix; out of which 48 neighboring pixels of each center pixel are grouped into 2 sets of 24 pixels each i.e., G1, G2, which is also equivalently termed as regions (i.e., region-1, region-2) [17]. Each of these groups is then further computed into LBP histogram and further to a joint histogram after combining resultant 2 LBP histograms. The histograms are computed by converting each LBP image into bins of size 26, which are numbered based on LBP codes ranging from 0 to 25. This has finally resulted into feature vector of size 52 per slice and finally resulted into feature set of size 260 for five selected slices of every subject.

### Feature selection

Dimensionality reduction and feature selection is performed using recursive feature elimination ranking algorithm. RFE can approximate the data using fewer variables while preserving as much information as possible. It fits a model and eliminates the features which are not useful until the specified number of features is reached [42], [43], [44], [45]. It also removes any dependencies or collinearity that may be present in the model. These top features are extracted in such a way that they are more important and these help to produce more accurate results.

Each of the variants has an individual algorithm to calculate the LBP features and LBP histogram. LBP variant-I with 8-bit LBP results in three LBP histograms per image which are merged together for five slices of a subject, that yields total 150 features. Likewise, LBP variant-II with 8-bit LBP produce a feature vector of size 300 for a subject by combining 60 LBP histogram features of five selected slices of the corresponding subject. It generates maximum number of LBP histograms in the output which results in maximum number of texture features as compared to other variants. Similarly, LBP variant-III with 16-bit LBP results in three LBP histograms per image which are merged together for five slices of a subject, that yields total 270 features and LBP variant-IV with 24-bit LBP generates two LBP histograms per image which are combined together for five slices of a subject, that produces total 260 features.

The current work has selected the features which had an importance score of greater than or equal to 0.9. Around 150 features, that are derived from LBP variant-I for a subject, are reduced to 15 features using RFE. Similarly, 300 features of variant-II are reduced to 17 features, RFE has selected 13 features among 270 features of variant-III, and 260 features were reduced to 21 features of LBP variant-IV. Table 1 shows the details about feature extraction in all variants.

**Table 1:** Number of selected features in all LBP variants using RFE.

LBP Variant, number of pixel groups	Number of LBP Histograms per slice, number of histogram features per slice,	Number of histogram features per subject (each subject with 5 slices)	Selected top features with importance score >=0.9 using RFE
Variant-I (8-bit LBP), three regions with each of 8 pixels	3, 30	150	15
Variant-II (8-bit LBP), six regions with each of 8 pixels	6, 60	300	17
Variant-III (16-bit LBP), three regions with each of 16 pixels	3, 54	270	13
Variant-IV (24-bit LBP), two regions with each of 24 pixels	2, 52	260	21

### Machine learning-based classification

Support vector machine is one of the popular supervised machine learning algorithms used for both classification and regression tasks, but mostly used in classification [46,47]. More accuracy with less computation is a major advantage of using SVM. It creates a hyperplane in *n*-dimensions to classify input points. Hyperplanes having maximum margin between both classes are considered for classification. In this study, a support vector classifier (SVC) instance is imported from sklearn.svm library. Kernel parameter of the SVC instance was set to the linear value and the gamma parameter to auto settings.

Random forest algorithm is also one of the common supervised algorithms [48,49], which is here used to classify between PD patients and control subjects. The random forest model was cross-validated on advanced biomedical textural markers extracted by using LBP variants. Collection of decision trees were used to build random forest classifiers. Each best output from decision trees is considered to analyse output of random forest. Sklearn library is used to import random forest classifiers with Python implementations. The number of decision trees were set to 150 and was decided using cross entropy for a more efficient model.

5. **Experimentations and Results**

Initially the dataset was in the base format of neuroimaging functional toolkit (NIfTY) (.nii files) which were read using the python library Nibabel. The data was in the form of 4D images where the first two dimensions represented the image pixel intensities, 3^rd^ and 4^th^ dimensions represented the slice number and time points respectively. These data were reformatted by taking the transpose of the image matrix to access it correctly. All four variants of LBP textures were applied to each slice of every brain of the dataset. These generated textures were transformed into its corresponding LBP histogram. Further, RFE was applied to reduce the dimensionality of data to select top features with importance scores of greater than 0.9. The selected feature set was fed variant wise to random forest and SVM models to cross-validate and test on independent cohorts. The parameters like different test and train size were used to split the dataset ranging from 10% to 50% of total size for testing cohort against training cohort. After this manual tuning, 25% of test size was decided as most appropriate due to its better performance and the same was used for final experimentation by holding out separate test cohort of 18 subjects (12 PD patients and 6 healthy subjects). 75% of training data was used to train the models with total 54 subjects (27 PD patients and 27 healthy subjects) using 10-fold cross validation. The performance measures such as accuracy, AUC-ROC, precision, recall and f1-score are calculated to evaluate the model.

**Fig. 5:**
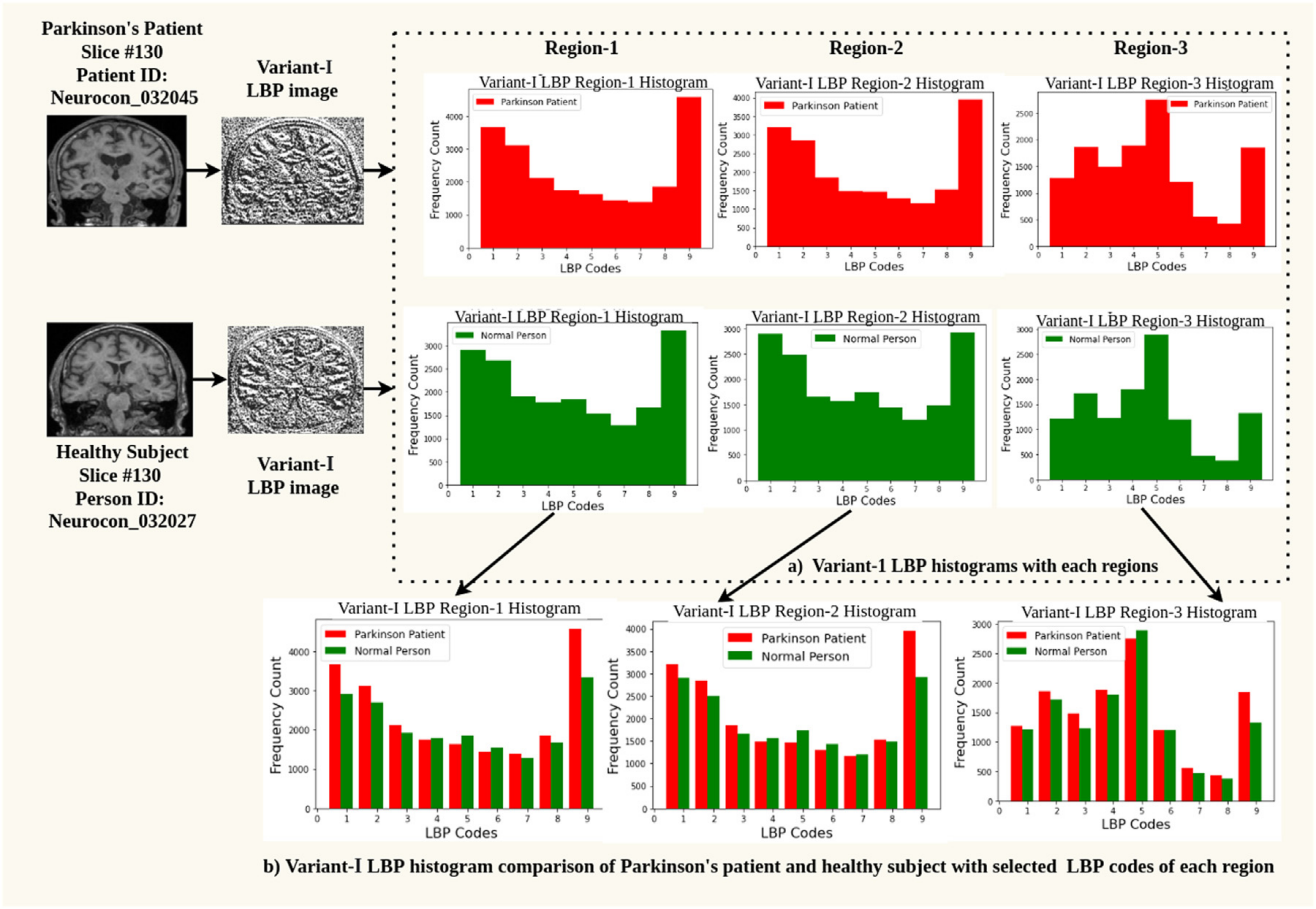
LBP histograms drawn using LBP variant-I on region-1, region-2, region-3 for sample Parkinson's patient and healthy control.

LBP histograms of region-1, region-2, region-3, that are drawn from LBP variant-I, are shown in Fig. 5 for sample subjects containing a healthy control and a Parkinson's patient.

The visual difference can be observed between histograms of Parkinson's patient vs healthy subjects. The difference is shown for variant-I of LBP which has comparatively resulted in better accuracy. It also shows an intermediate image which is the texture generated by the LBP algorithm and the histograms are extracted from these textures. There is a difference between LBP codes of '1′ to '9′ between PD and normal subjects, which contribute to top performing features.

**Fig. 6:**
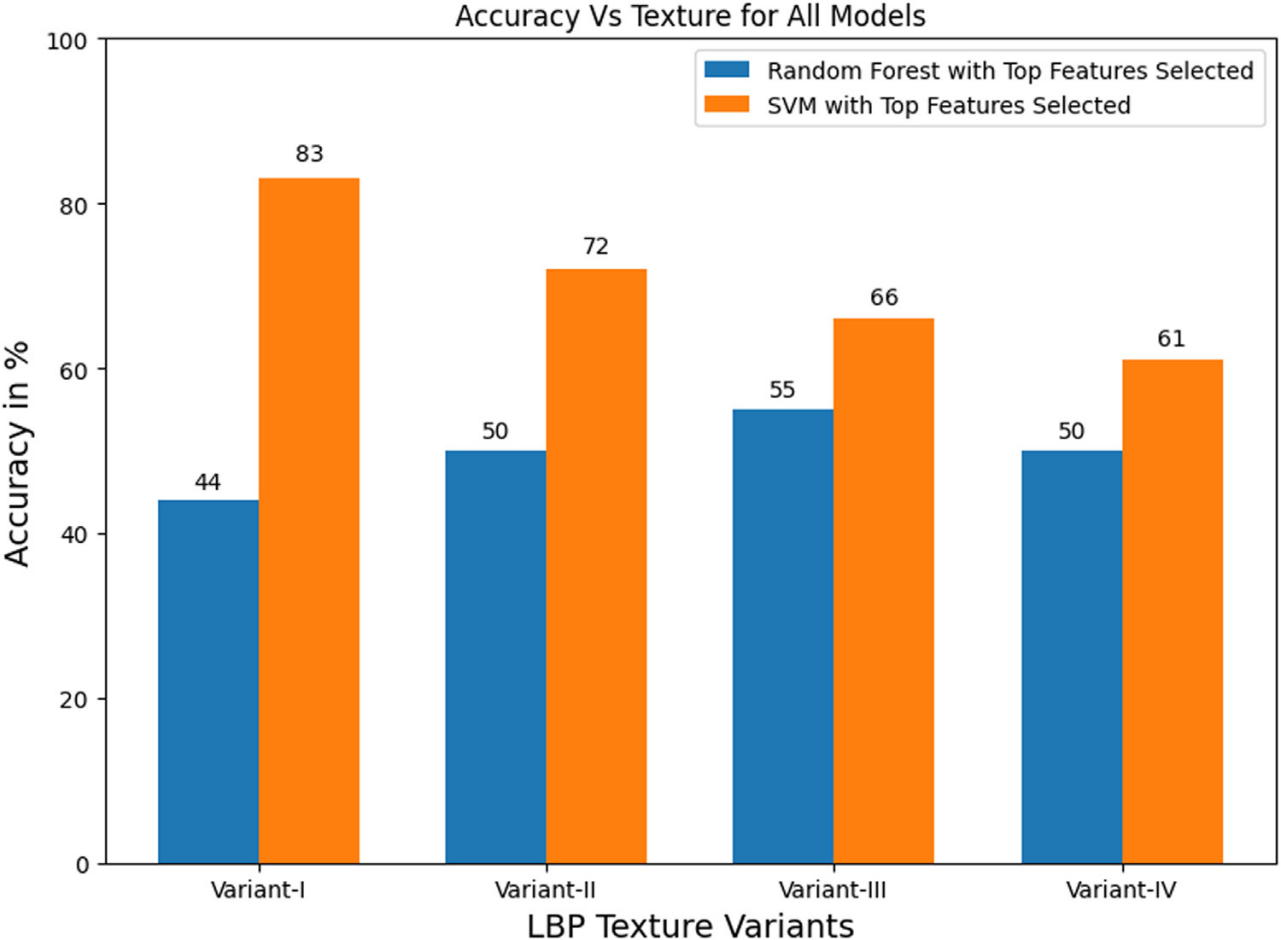
Accuracies of all LBP variants using SVM and random forest.

The accuracies obtained with feature selection and without feature selection using SVM method and the accuracies obtained with feature selection using random forest method are shown in Fig. 6. These metrics are shown for all LBP variants.

Fig. 6 shows best performance of LBP variant-I using SVM classifier with test accuracy of 83.33% with the selected top 15 features. The model of SVM performed well for all variants as compared to Random Forest. Also, SVM model with RFE-based feature selection has worked better as compared to SVM model without feature selection.

Among all variants, variant-I has obtained maximum test accuracy of 83.33% using a selected feature set of size 15 and using a support vector machine classifier. It has achieved precision of 84.62%, recall of 91.67%, f1-score of 88% and AUC of value 0.86 during testing phase. The performance of all four LBP variants using SVM method is summarized in Table 2.

**Table 2:** Summary of performance scores of all LBP variants using SVM.

LBP variant	Cross-validation metrics		Testing metrics				
	Training accuracy (%)	Validation accuracy (%)	Testing accuracy (%)	Precision	Recall	f1-Score	AUC
Variant-I (8-bit LBP with 3 sets of 8 pixels in 5 × 5 neighborhood)	87.01	71.33	83.33	0.84	0.91	0.88	0.86
Variant-II (8-bit LBP with 6 sets of 8 pixels in 7 × 7 neighborhood)	87.03	66.33	72.22	1.0	0.58	0.73	0.76
Variant-III (16-bit LBP with 3 sets of 16 pixels in 7 × 7 neighborhood)	79.44	63.00	66.67	0.75	0.75	0.75	0.43
Variant-IV (24-bit LBP with 2 sets of 24 pixels in 7 × 7 neighborhood)	66.27	53.67	61.11	0.69	0.75	0.72	0.74

**Fig. 7:**
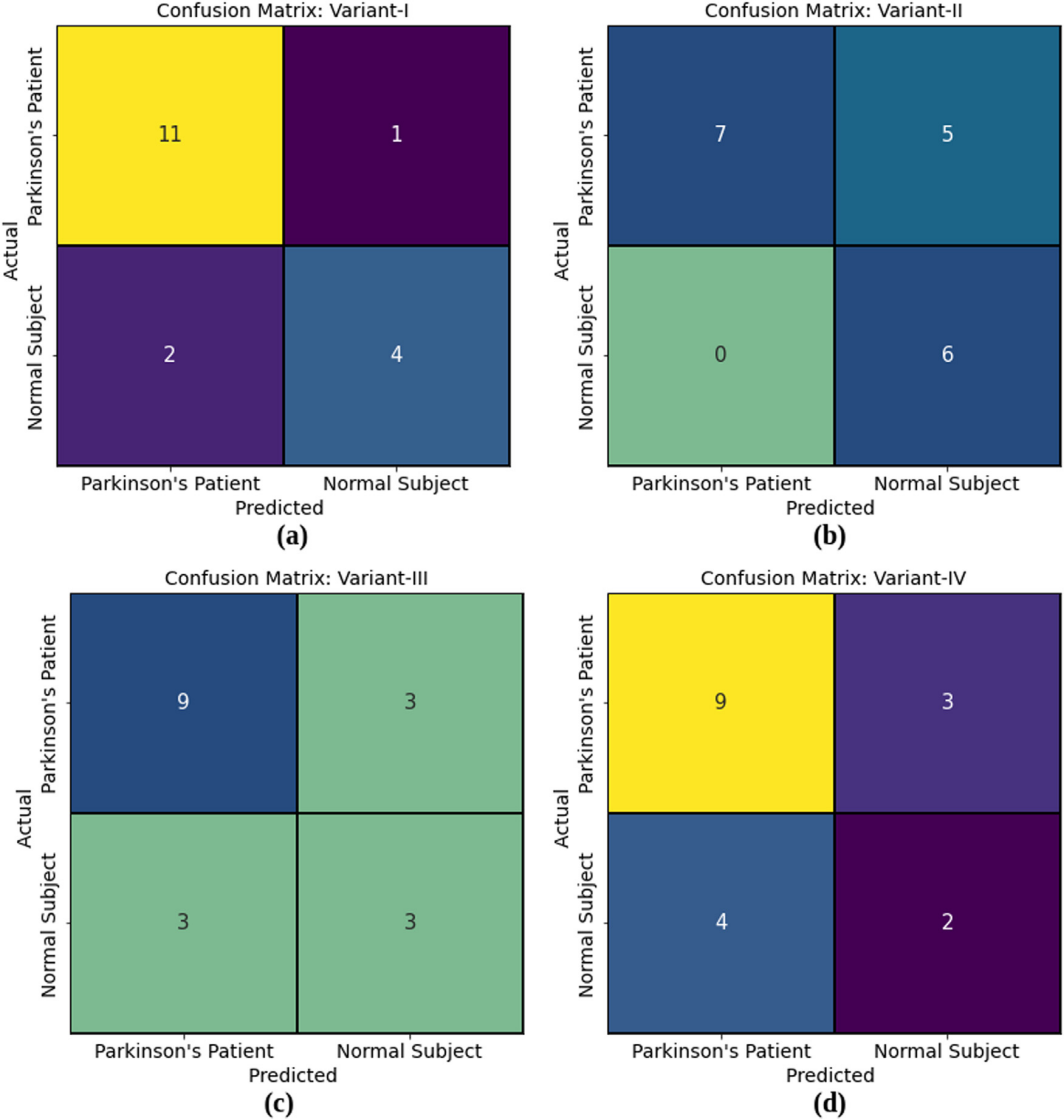
Confusion matrix (a) Variant-I; (b) Variant-II; (c) Variant-III; (d) Variant-IV.

Confusion matrices of radiomic model using SVM and all four LBP variants are shown in Fig. 7.

**Fig. 8:**
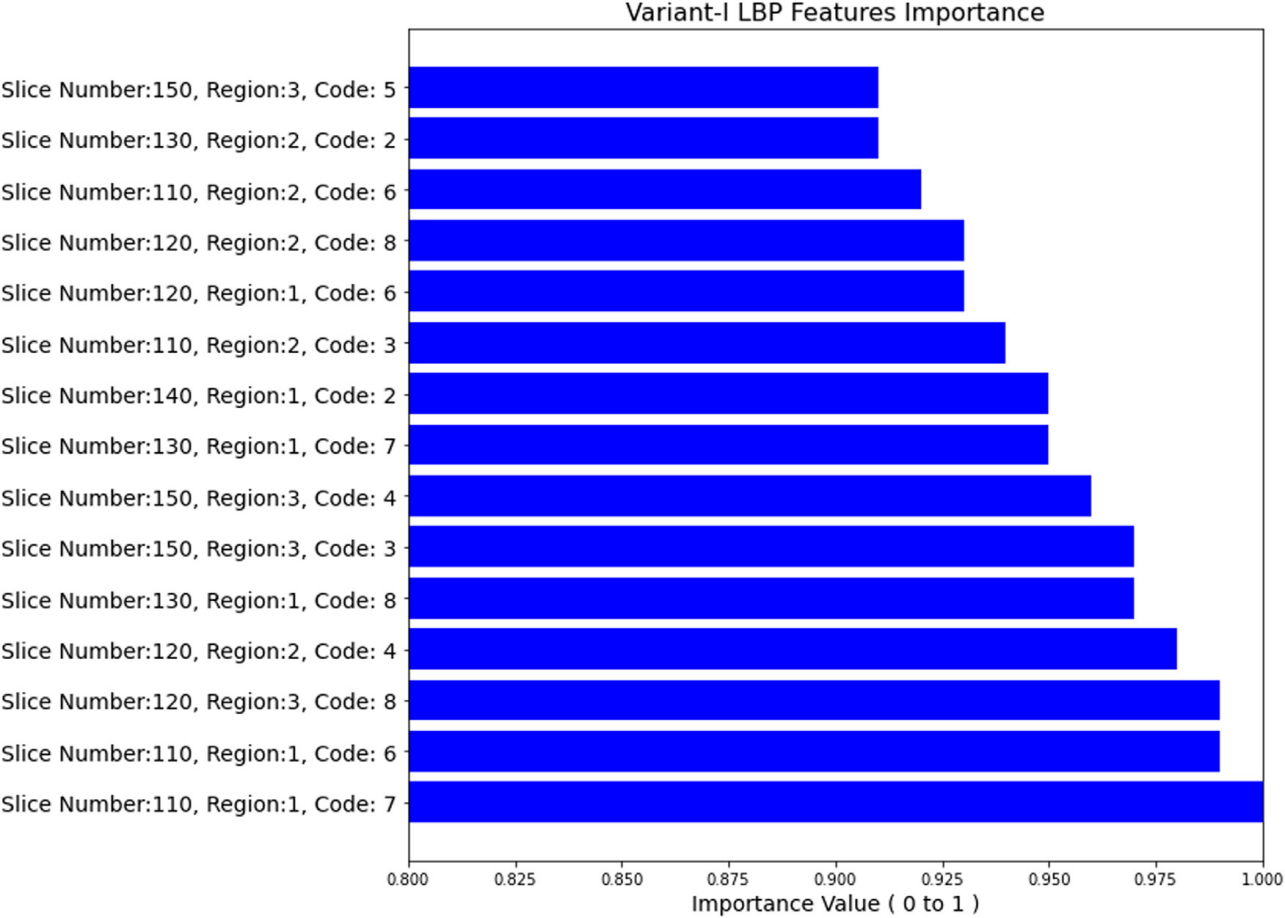
Top features selected using RFE from LBP variant-I derived feature set.

The selected top features of variant-I are displayed in graph in Fig. 8. The *x*-axis represents importance score value and variant-I-derived top features are presented on *y*-axis. Higher the score, higher the importance of a particular feature. The features on *y*-axis are displayed in an elaborated manner like the slice number of a subject from which the feature is extracted, region number among three regions of 8 pixels each of variant-I, LBP code number in decimal derived using rotation-invariant uniform LBP.

**Fig. 9:**
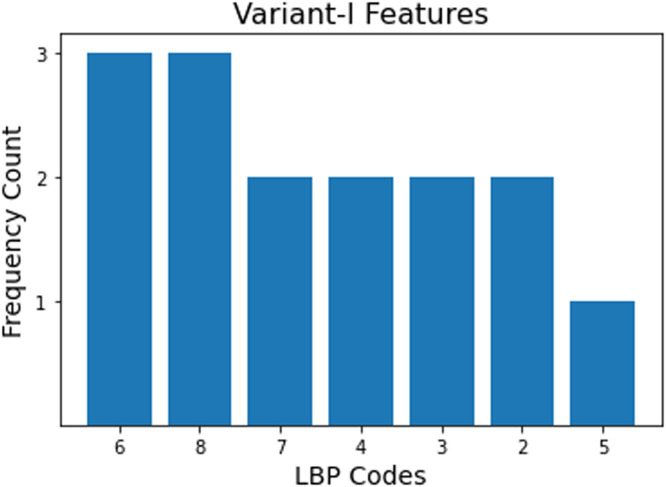
Frequency count of top LBP codes of variant-I histogram.

It can be observed from Fig. 8 that the most significant feature is LBP code '7′ which has an importance score of 1. It also signifies that LBP codes in region-1 can contribute to produce more appropriate results. It is also observed from Fig. 9, 8^th^ and 6^th^ LBP code is repeated the most with 3 times for variant-I in the list of top selected features. According to rotation-invariant uniform LBP method, LBP code ‘7’, ‘6’, ‘4’ generally represents edges. So, Fig. 9 demonstrates that there are certain variations in local patterns (in term of edges) of both the classes (PD patient and normal subject).

**Fig. 10:**
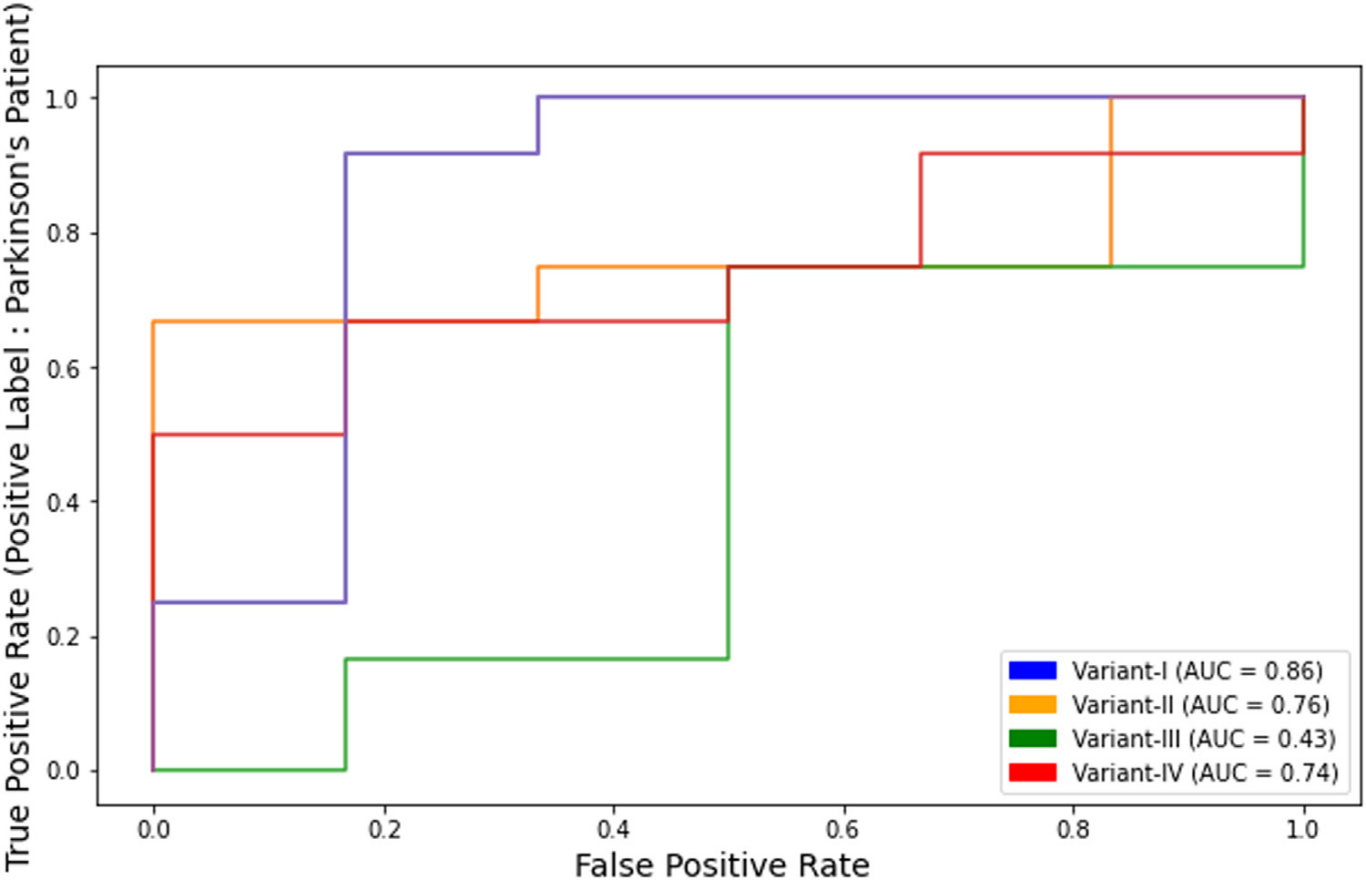
AUC-ROC of LBP variants for Parkinson's prediction with feature selection.

The AUC-ROC curve of all variants with RFE feature selection is shown in Fig. 10. These graphs are plotted with true positives and false positives for labels (such as 'Parkinson's patient' and 'healthy subject') in PD classification. Fig. 10 shows the maximum AUC-ROC for variant-I with a value of 0.86.

The proposed method is compared with the existing methods as shown in Table 3.

**Table 3:** Comparison of proposed method with existing methods.

Dataset details	Method	Feature set	Testing Accuracy/precision /recall/ sensitivity /AUC-ROC/ specificity
PPMI (3T T1 MRI scans with 3D images, 906 subjects) [30]	Support vector machine	Textural, morphological, and statistical features	81.74% accuracy, 82.41% precision, 80.71% recall, 81.78% f1-score
MRI scans – structural & functional, 213 subjects) [37]	Support vector machine	Intensity histogram, texture, wavelet	78.07% accuracy, 78.80% sensitivity, 76.08% specificity, 0.85 AUC
MRI Dataset (T1- weighted images, 103 subjects) [50]	Support vector machine	Morphological features	65% accuracy, 66.7% precision, 62.2% recall, 0.69 AUC
MRI dataset (Resting state images, 120 subjects) [51]	Random forest	Regional homogeneity, resting-state functional connectivity and gray matter volume	82.61% accuracy 0.90 AUC
MRI images- susceptibility weighted imaging (SWI) images, 190 subjects) [52]	Random forest	Intensity, shape, gray level co-occurrence matrix, run length matrix, gray level size zone matrix features	69% accuracy, 73% specificity 64% sensitivity
Proposed method (LBP variant-I)	Support vector machine	Advanced LBP histogram features	83.33% accuracy, 84.62% precision, 91.67% recall, 88% f1-score, 0.86 AUC

It is demonstrated with the current study that more expressive local patterns are recognized by LBP variant-I method using small number of neighboring pixels with small scale. Therefore, variant-I with 8-bit LBP method is relatively more proficient to distinguish the elusive variations between the classes with PD and without PD.

## Conclusion

The current work has carried out the radiomics-based classification of Parkinson's disease using advanced biomedical textural features by applying LBP variants. Four LBP variants, that includes 8-bit LBP on 5 × 5 neighborhood with 3 histograms, 8-bit LBP on 7 × 7 neighborhood with 6 histograms, 16-bit LBP on 7 × 7 neighborhood with 3 histograms and 24-bit LBP on 7 × 7 neighborhood with 2 histograms, were used for extraction of most distinguishing MRI-derived textures to differentiate Parkinson's patient and healthy subject. Feature selection has achieved better performance accuracy of 83.33% using LBP variant-I and SVM. Number of features was reduced to 15 features, 17 features, 13 features and 21 features for LBP variant-I to variant-IV respectively using the RFE algorithm. Further evaluation parameters like accuracy, precision, recall, and f1 score also showed great results for variant-I as well as for variant-II of LBP textures. The texture analysis provided the more accurate feature extraction technique as compared to normal LBP models. This method will be helpful for automating the task of PD classification which was difficult to detect using manual diagnosis.

## CRediT authorship contribution statement

**Sonal Gore:** Conceptualization, Methodology, Writing – original draft, Investigation, Project administration. **Aniket Dhole:** Data curation, Formal analysis, Resources, Software. **Shrishail Kumbhar:** Data curation, Formal analysis, Resources, Software. **Jayant Jagtap:** Supervision, Validation, Visualization, Writing – review & editing.

## Declaration of Competing Interest

The authors declare that they have no known competing financial interests or personal relationships that could have appeared to influence the work reported in this paper.

## Data Availability

Data is publicly available.
